# Outcomes for depression and anxiety in primary care and details of treatment: a naturalistic longitudinal study

**DOI:** 10.1186/1471-244X-11-180

**Published:** 2011-11-18

**Authors:** Marijn A Prins, Peter FM Verhaak, Mirrian Hilbink-Smolders, Peter Spreeuwenberg, Miranda GH Laurant, Klaas van der Meer, Harm WJ van Marwijk, Brenda WJH Penninx, Jozien M Bensing

**Affiliations:** 1 NIVEL, Netherlands Institute for Health Services Research, (Postbus 1568), Utrecht, (3500 BN), the Netherlands; 2 Dep. of General Practice, University Medical Centre Groningen, (Postbus 30001), Groningen (9700 RB), the Netherlands; 3 Scientific Institute for Quality of Healthcare, Radboud University Nijmegen Medical Centre, (Postbus 9101, 114) Nijmegen, (6500 HB) the Netherlands; 4 Department of General Practice, VU University Medical Center, (Postbus 7057), Amsterdam (1007 MB), the Netherlands; 5 Department of Psychiatry/EMGO Institute/Neuroscience Campus Amsterdam, VU University Medical Center, (A.J. Ernststraat 887) Amsterdam, 1081 HL the Netherlands; 6 Department of Psychiatry, Leiden University Medical Center, (Postbus 9600), Leiden, (2300 RC), the Netherlands; 7 Department of Psychiatry, University Medical Center Groningen, (Postbus 11120), Groningen, (9700 CC), the Netherlands; 8 Department of Clinical and Health Psychology, Utrecht University, (Postbus 80140), Utrecht, (3508 TC), the Netherlands

## Abstract

**Background:**

There is little evidence as to whether or not guideline concordant care in general practice results in better clinical outcomes for people with anxiety and depression. This study aims to determine possible associations between guideline concordant care and clinical outcomes in general practice patients with depression and anxiety, and identify patient and treatment characteristics associated with clinical improvement.

**Methods:**

This study forms part of the Netherlands Study of Depression and Anxiety (NESDA).

Adult patients, recruited in general practice (67 GPs), were interviewed to assess DSM-IV diagnoses during baseline assessment of NESDA, and also completed questionnaires measuring symptom severity, received care, socio-demographic variables and social support both at baseline and 12 months later. The definition of guideline adherence was based on an algorithm on care received. Information on guideline adherence was obtained from GP medical records.

**Results:**

721 patients with a current (6-month recency) anxiety or depressive disorder participated. While patients who received guideline concordant care (N = 281) suffered from more severe symptoms than patients who received non-guideline concordant care (N = 440), both groups showed equal improvement in their depressive or anxiety symptoms after 12 months. Patients who (still) had moderate or severe symptoms at follow-up, were more often unemployed, had smaller personal networks and more severe depressive symptoms at baseline than patients with mild symptoms at follow-up. The particular type of treatment followed made no difference to clinical outcomes.

**Conclusion:**

The added value of guideline concordant care could not be demonstrated in this study. Symptom severity, employment status, social support and comorbidity of anxiety and depression all play a role in poor clinical outcomes.

## Background

Depression and anxiety are common mental disorders which cause considerable emotional and physical suffering, often resulting in severe disability [1-5]. Primary care settings have become the principal site for treating depressive and anxiety disorders [3,6] and quality of care for anxiety and depression seems to be moderate or poor [7-10].

Over the past decade, many evidence-based guidelines have been developed [11]. However, little is known about the effects of their application on clinical care outcomes [12]. Implementation of evidence-based clinical guidelines has been advocated as a way of improving detection and treatment of common mental disorders and reducing variations in health care [13]. Guidelines specify low and high intensity psychological and pharmacological interventions with proven effectiveness. A stepped care approach (preference for the least restrictive and least costly interventions) has been advocated. Collaborative care (integration of generalist and specialist care) is a critical element in the latest versions [14]. In the Netherlands, the Dutch College of General Practitioners (DCGP) issued evidence-based general practice guidelines for depression and anxiety [15,16], which are widely accepted and play a prominent role in continuing professional development programmes for medical practitioners[17]. These guidelines follow the international accepted state of the art and are comparable with British [14] and American [18] guidelines.

There is some evidence that guideline concordant treatment is positively associated with improvements in patients with depressive [19] and anxiety disorders [20]. However, randomised controlled trials designed to improve outcomes for anxiety and depression in primary care, by structured implementation of evidence-based guidelines, show mixed results [21]. In addition, systematic reviews report little effect of guideline implementation [12,22]. The Hampshire Depression Project, a major trial on implementing guideline concordant care, could not show improvements in diagnosis of or recovery from depression [23]. Croudace et al. [24] did not find an effect of guideline implementation on detection and outcome for mental disorders either. However, these studies did not analyse patient characteristics regarding their possible benefit from guideline concordant care. Furthermore, no distinction was made between the various types of care (psychological interventions, pharmacological interventions or referral).

Although clinical severity and treatment adequacy play a role in symptomatic improvement and full recovery from a depressive episode, recovery also seems to be influenced by social support, education level, age, (un)employment and non-depressive psychopathology [25-29]. For anxiety disorders, a good outcome was predicted by mild symptoms, high education level and being employed, as well as male gender and later onset [25,30,31], while comorbidity with major depression worsened clinical outcomes in a 12-year study [32].

Therefore, whether or not guideline concordant care in general practice will improve clinical outcomes in anxiety and depression patients *with specific characteristics or with specific interventions *has yet to be demonstrated. Consequently, the following questions will be addressed:

1) Do primary care patients with a current anxiety or depressive disorder, who received guideline concordant care, show greater clinical improvement after one year than patients who did not receive care in accordance with the guidelines?

2) Which patient characteristics are associated with particular clinical outcomes after one year?

3) Which interventions are associated with particular clinical outcomes after one year?

## Methods

### Setting and recruitment

Data were collected in the Netherlands Study of Depression and Anxiety (NESDA, http://www.nesda.nl). NESDA is a multi-site naturalistic cohort study designed to measure the long-term course and consequences of depressive and anxiety disorders [33]. For this study, primary care data were used.

**Figure 1:**
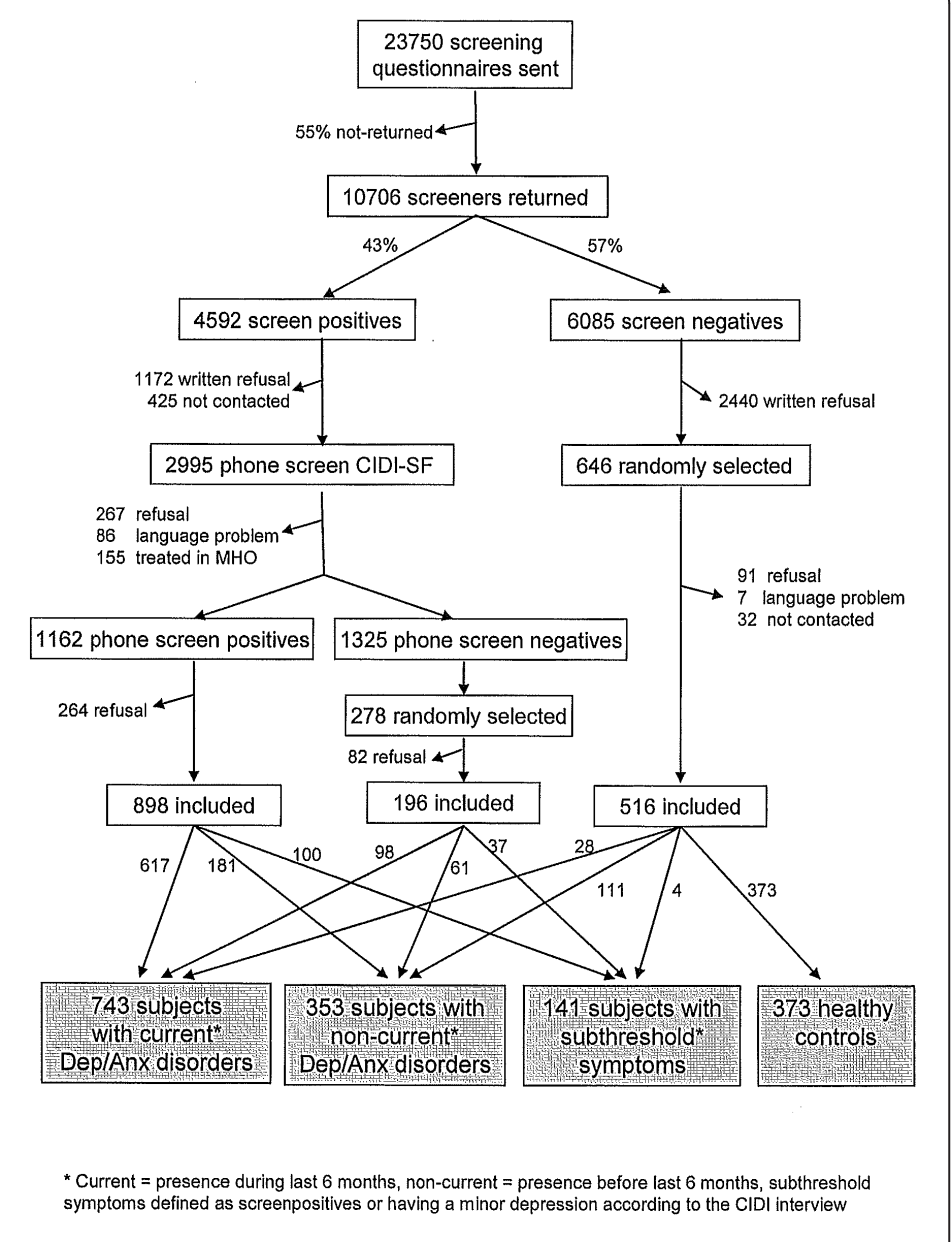
**Recruitment flow of NESDA respondents in the primary care sample**.

Adult patients (18-65 years old) were recruited from 67 GPs (21practices), selected based on their use of electronic medical record (EMR) systems. Patients who attended their GP in the last 4 months, irrespective of the reason for consultation, were sent a questionnaire consisting of the Kessler-10 (K-10) [34], with five additional questions to screen for depressive or anxiety disorders. Nearly half of the questionnaires returned were screen-positive (K-10 score of 20 or higher or a positive score on any of the additional anxiety questions). Women and older people were more likely to return the questionnaire, but there were no differences in psychopathology between responders and non-responders [33,35]. These people were interviewed by phone with the short form of the Composite Interview Diagnostic Instrument (CIDI). Patients who fulfilled the CIDI-short form criteria for a current (6-month recency) depressive or anxiety disorder, were asked to participate in NESDA and were invited for a baseline assessment. For a detailed summary of the sampling procedure, see Figure 1.

### Measurements

During baseline assessment, all patients were interviewed and completed questionnaires to collect detailed socio-demographic data including age, gender, education level (3 levels), employment status, income, marital and partnership status, and personal network size. The CIDI interview, WHO Lifetime Version 2.1 was conducted which identified 743 patients who met DSM-IV criteria [36] for a current depressive (Major Depressive Disorder (MDD), dysthymia) or anxiety disorder (generalised anxiety disorder, social phobia, panic disorder, agoraphobia). Since 22 patients refused to give informed consent for the use of their EMR, ultimately 721 patients were included in this study.

One year after inclusion, each participant received a questionnaire containing the most important self-report instruments (measuring severity of depression and anxiety, see below) to determine the course of anxiety and depression symptoms.

### Actual GP care: guideline concordant care versus non-guideline concordant care

Information on the delivery of care given to patients with a current anxiety or depression diagnosis (as measured by the CIDI) was gathered from GPs' EMRs. Data were extracted from the year prior to inclusion in the NESDA study to one year after inclusion. The following data were collected for each patient included in the study,: number and type of contacts, International Classification of Primary Care (ICPC) codes [37], prescribed medication (type and dose), duration of prescription, and referrals. Our earlier NESDA paper [38] described the degree to which GPs adhered to the evidence-based DCGP clinical depression and anxiety guidelines in the delivery of care for their anxiety and depression patients [15,16]. Based on the care they had received, patients were divided into two groups, i.e. patients who had received 1) guideline concordant care, or 2) non-guideline concordant care. The algorithm for guideline concordant care is:

› **PSYCHOLOGICAL SUPPORT including at least five consultations in the 15 weeks following documentation of diagnosis**

OR

› **COUNSELLING (only applicable to GP depression care)**

OR

› **PRESCRIPTION OF ANTIDEPRESSANT MEDICATION including evaluation after six weeks of prescription and minimal duration of five months or cessation in case of no response**

OR

› **REFERRAL TO MENTAL HEALTH SPECIALIST**

Patients fulfilling one of the criteria (receiving psychological support, counselling, AD-medication or referral, according to the specifications given above) are considered to have received guideline concordant care. 50% of patients with depression and anxiety disorders received guideline concordant care, mainly counselling, medication or referral. 42% of patients with depression only received guideline concordant care, mainly in the form of counselling or referral. Only 27% of patients with anxiety disorders only received guideline concordant care, mainly in the form of referral.

This algorithm is necessarily a simplified version of treatment recommendations described in various guidelines. It only takes account of whether a treatment is in place, without including the content of the interventions or the conditions under which treatment occurs. This is due to lack of data e.g. regarding severity of symptoms during GP contact or treatments already tried in the past.

Patients fell into the non-guideline concordant care group if they did not fulfil any of the abovementioned criteria (i.e. if they did not receive counselling, sufficient pharmacological treatment or referral).

### Clinical outcome measures

Clinical status was measured by the 30-item Inventory of Depressive Symptoms self-report (IDS-SR), which measures the severity of depressive symptoms, and has shown highly acceptable psychometric properties [39], as well as the 21-item Beck Anxiety Inventory (BAI), measuring anxiety symptoms [40]. Total scores of the IDS-SR and BAI could range from 0 to 84 and 0 to 63 respectively, where high scores indicate more (severe) symptoms. These clinical outcome measures were measured at the baseline assessment of NESDA (T0) and at one year follow-up (T1).

### Statistical analyses

First, χ^2 ^and *t*-test for independent samples were used to compare patient characteristics of those who received guideline concordant care with those who received non-guideline concordant care.

Secondly, using MLwiN software, a multilevel repeated measures analysis was undertaken to test whether improvements in symptom severity were statistically significantly different between follow-up and baseline. Since data were grouped (clustered) by GPs and practices, a random intercept was included in the model to adjust for possible differences resulting from this clustering. All values were corrected for age, gender, education level and baseline severity score. The multilevel model takes all available data into account (the paired samples that had completed the questionnaires on both occasions, as well as the unpaired data of those patients who only completed the questionnaires at baseline). For the outcome measures on baseline and follow-up, adjusted means and standard errors were calculated for both treatment groups. To compare differences in trends from baseline to follow-up between the two care groups, differences in means were tested using Wald statistics (df = 1). Trends were considered significant if Chi-square was > 3.85 (P < .05).

Thirdly, multilevel regression analyses were performed to model associations between socio-demographic characteristics, severity scores at baseline and specific types of treatment (counselling/psychological support, antidepressants, referral), with clinical severity at follow-up as the outcome variable. Fixed and random parameter estimates and their standard errors (SE) were calculated.

Finally, multilevel logistic regression analyses were performed to model associations between different patient characteristics and (still) having moderate or severe anxiety or depressive symptoms at follow-up. Socio-demographic characteristics were entered, followed by social support variables and type of diagnosis. In the last step, severity symptoms at baseline were added to the model.

### Ethical approval

The NESDA study was approved centrally by the Ethics Review Board of the VU University Medical Center and by local review boards of the participating institutes. Following the provision of verbal and written information on the study, written informed consent was obtained from all participants.

## Results

### Patient characteristics: guideline concordant care versus non-guideline concordant care

As described elsewhere [34], 281 (39%) of patients received guideline concordant care from their GP and 440 (61%) patients received non-guideline concordant care. There were no significant differences between the two groups with regard to gender, age, marital status, working status and education level (Table 1). However, patients who received guideline concordant care were more likely to suffer from comorbidity of both anxiety and depressive disorders, than they were to suffer from an anxiety disorder only, compared with patients who received non-guideline concordant care. At baseline, patients who received guideline concordant care had on average significantly higher severity scores than the non-guideline concordant care group.

**Table 1:** Comparisons of patients who received Guideline concordant care and Non-guideline concordant care at baseline (T0)

	Guideline concordant care, N = 281	Non-guideline concordant care, N = 440	P-value
Female gender (%)	68.0	71.6	0.30

Age in years, mean (SD)	45.0 (11.4)	44.9 (12.5)	0.89

Married/living together (%)	44.5	38.4	0.11

Working (%)	64.4	66.4	0.59

Education level (%)			0.22
Basic	7.1	10.9	
Intermediate	60.9	57.3	
High	32.0	31.8	

Type of diagnosis (%)			
Anxiety disorder(s) only	28.8	49.3	< 0.0001
Depressive disorder(s) only	22.1	19.5	0.41
Comorbidity of both depressive and anxiety disorders	49.1	31.1	< 0.0001

Clinical outcome measures T0, mean (SD)			
Severity of depressive symptoms (IDS-SR)	30.8 (12.1)	25.3 (10.7)	< 0.0001
Severity of anxiety symptoms (BAI)	17.9 (11.3)	15.1 (9.5)	0.001

At follow-up, 139 patients (19%) had been lost as a result of attrition. Compared with non-completers, completers were older (45.7 vs. 41.5), more highly educated, and reported less severe anxiety (15.2 vs. 20.5) and depression (26.6 vs. 30.9) symptoms.

### Clinical outcome and guideline adherence

The adjusted means and standard errors for the guideline concordant and non-guideline concordant care groups for the outcome measures on T0 and T1 are presented in Table 2 for patients with at least one depressive disorder, and in Table 3 for patients with at least one anxiety disorder. Patients with depression improved significantly on depressive symptoms between T0 and T1, and patients with anxiety improved on their anxiety symptoms (P < .001). After controlling for patients' age, gender, education level, baseline severity score and clustering, patients from the non-guideline concordant care group had improved just as much as those from the guideline concordant care group in both depression (6.5 resp. 8.1 points) and anxiety (2.9 resp. 4.0) levels.

**Table 2:** Differences in severity of depressive symptoms in patients with depression (N = 423) who received guideline concordant care (GCC) versus non-guideline concordant care (NGCC) at baseline (T0) and after 12 months (T1)

Outcome measure	T0		T1		Δ T0-T1
	GCC	NGCC	GCC	NGCC	GCC NGCC

IDS score, mean	32.9 (0.9)^a^	28.5	24.8 (1.0)^a^	22.0	8.1^c ^
(SD)	(0.8)^b ^		(0.8)^ b^		6.5^c^

^a ^GCC: T0 > T1 (χ^2 ^= 68.5; p < .001)

^b ^NGCC: T0 > T1 (χ^2 ^= 64.9; p < .001)

^c ^ΔT0-T1: ns

All values are corrected for age, gender, education level and baseline severity score, and clustering within practices and GPs.

**Table 3:** Differences in severity of anxiety symptoms in patients with anxiety (N = 573) who received guideline concordant care (GCC) versus non-guideline concordant care (NGCC) at baseline (T0) and after 12 months (T1)

Outcome measure	T0		T1		Δ T0-T1	
	GCC	NGCC	GCC	NGCC	GCC NGCC	

BAI score, mean	18.7 (0.8)^a^	15.1	14.7 (0.8)^a ^	12.2	4.0^c^	2.9^c^
(SD)	(0.6)^b^		(0.5)^b^			

^a ^GCC: T0 > T1 (χ^2 ^= 27.8; p < .001)

^b ^NGCC: T0 > T1 (χ^2 ^= 26.7; p < .001)

^c ^ΔT0-T1: ns

All values are corrected for age, gender, education level and baseline severity score, and clustering within practices and GPs.

### Associations with socio-demographics and type of treatment

Associations between severity of symptoms at T1 and socio-demographics and type of treatment are presented in Table 4.

**Table 4:** Multilevel regression analysis on severity of depressive and anxiety symptoms respectively at T1 by patient characteristics and type of treatment received in patients with depressive (N = 322) and anxiety disorders (N = 457)

	Estimate (SE)	P-value
**Patients with depressive disorder(s)**		

Severity of depressive symptoms at T0	0.61 (0.05)	< 0.0001

Female gender^a^	0.85 (1.17)	0.47

Age	0.11 (0.05)	0.01

Basic education level^b^	3.59 (2.12)	0.09
Intermediate education level^b^	2.21 (1.12)	0.05

Counselling received	1.47 (1.51)	0.33

Antidepressants received for ≥ 5 months	-2.87 (2.16)	0.18

Referred to a mental health specialist	0.84 (1.31)	0.52

**Patients with anxiety disorder(s)**		

Severity of anxiety symptoms at T0	0.58 (0.04)	< 0.0001

Female gender^a^	-0.23 (0.78)	0.76

Age	0.03 (0.03)	0.27

Basic education level^b^	2.01 (1.37)	0.14
Intermediate education level^b^	1.79 (0.78)	0.02

Psychological support received	-1.43 (2.08)	0.49

Antidepressants received for ≥ 5 months	-0.98 (1.59)	0.54

Referred to a mental health specialist	0.30 (0.89)	0.74

^a ^reference category = male gender; ^b ^reference category = high education level

All values are corrected for clustering within practices and GPs.

Severity of depressive and anxiety symptoms respectively at T1 (dependent variables) was significantly associated with severity score at baseline and with intermediate (versus high) education level both for patients with depressive disorders and patients with anxiety disorders. In patients with depressive disorders, higher age was also positively associated. No significant associations between any of the different care forms, for either depression or anxiety, and clinical outcomes at T1 were found.

### Patient characteristics and course of anxiety and depression

Patients who (still) had moderate or high anxiety or depressive symptoms at follow-up (N = 213), were more likely to be unemployed and have lower income than patients who had low anxiety and depressive symptoms at follow-up (N = 364) (Table 5). When social support variables were added to the model, personal network size was significantly associated (OR = 0.94) with the severity of symptoms at follow-up. When the diagnosis was added, the presence of either anxiety disorders or depressive disorders without comorbidity decreased the chance of having moderate or severe symptoms after one year (comorbidity of both anxiety and depression increased this chance). However, when severity scores were finally added, all former associations disappeared and only the severity of depressive symptoms was significantly associated with clinical outcome; more severe depressive symptoms at baseline increased the chance of still having moderate or severe symptoms at follow-up. Thus, patients in adverse circumstances (unemployed, low income, small network) were more likely to retain a moderate/high symptom level at follow-up, because these were associated with higher baseline severity, which was the best predictor for lack of recovery at T1.

**Table 5:** Multilevel logistic regression analysis on (still) having moderate or severe depressive or anxiety symptoms at T1 (versus having low anxiety and depressive symptoms at T1)

	*Step 1*	*Step 2*	*Step 3*	*Step 4*
	**OR (95%- CI)**	**OR (95%- CI)**	**OR (95%- CI)**	**OR (95%- CI)**

*Socio-demographic variables*				

Female gender^a^	1.09 (0.73-1.62)	1.09 (0.73-1.62)	1.05 (0.70-1.58)	1.03 (0.65-1.64)

Age	1.01 (1.00-1.03)	1.01 (1.00-1.03)	1.02 (1.00-1.03)	1.02 (1.00-1.04)

Basic education level^b^	1.74 (0.86-3.52)	1.59 (0.77-3.28)	1.54 (0.73-3.25)	1.00 (0.42-2.35)
Intermediate education level^b^	**1.50 **(1.01-2.22)	1.47 (0.98-2.21)	1.50 (0.99-2.29)	1.45 (0.89-2.37)

Working	**0.56 **(0.38-0.84)	**0.56 **(0.38-0.84)	**0.64 **(0.42-0.97)	0.74 (0.46-1.19)

Income above modal (>2400 euro per month)^c^	**0.66 **(0.44-0.98)	0.69 (0.44-1.07)	0.70 (0.44-1.10)	0.68 (0.40-1.16)

*Social support variables*				

Married/living together		0.92 (0.58-1.47)	0.84 (0.52-1.36)	0.87 (0.50-1.52)

Having a partner		1.09 (0.68-1.76)	1.09 (0.67-1.78)	1.17 (0.67-2.05)

Personal network		**0.94 **(0.91-0.98)	**0.95 **(0.92-0.99)	0.98 (0.94-1.02)

*Type of diagnosis*				

Anxiety disorder(s) only^d^			**0.42 **(0.28-0.63)	1.27 (0.76-2.13)
Depressive disorder(s) only^d^			**0.37 **(0.22-0.63)	0.79 (0.43-1.44)

*Severity *				

Severity of symptoms at T0				
Depressive symptoms				**1.11 **(1.08-1.14)
Anxiety symptoms				1.03 (1.00-1.06)

OR in **bold **if P < 0.05.

^a ^reference category = male gender; ^b ^reference category = high education level; ^c ^reference category = less than modal (< 2400 euro per month); ^d ^reference category = comorbidity of both anxiety and depressive disorders.

## Discussion

### Summary of main findings

In this study we determined the possible associations between guideline concordant care and clinical outcomes in patients with anxiety and depressive disorders and identified patient and treatment characteristics associated with better or worse outcomes. Patients who were not treated in accordance with the general practice guidelines improved on their anxiety and depressive symptoms just as much as patients who were treated in accordance with those guidelines. While patients with comorbidity of both anxiety and depressive disorders, those with smaller personal networks and the unemployed were more likely to suffer from moderate or severe symptoms after 12 months. Severity of depressive symptoms at baseline was most strongly associated with severity after one year. In the case of depression, older patients and patients with an intermediate education level (as opposed to a high education level) had more severe symptoms after a year. In the case of anxiety, only education level was associated with severity. Different kinds of treatment did not result in different outcomes.

### Strengths and limitations of this study

The large sample size and the use of a prospective design in the collection of data to assess guideline adherence constitute the strong points of this study. The primary care sample of NESDA is representative to other GP attendees in the Netherlands and data for the same patient group were gathered at baseline and at the 12 month follow-up, which facilitated longitudinal comparisons. Furthermore, our data could be considered representative for "real life" treatment of depression and anxiety in primary care.

However, the naturalistic design of the study constitutes a major limitation. Participants could not be randomised and baseline scores differed markedly between the treatment groups. Even though estimated means were corrected for scores at baseline, it is still difficult to determine whether non-guideline concordant care is just as effective as care in accordance with the guidelines, or that differences between the predefined subgroups interfered with our results. Only a randomised controlled trial can directly test and compare the effectiveness of strategies that make care more guideline concordant. Finally, our classification into guideline concordant and non-guideline concordant care was based on available EMR data, which means that the quality of registration could have influenced our independent variables.

The study was conducted in the Netherlands, which constitutes a limitation because the (mental) health care system in the Netherlands differs in some respects from those in other countries. The position of the GP in the Dutch health care system is rather prominent, because he acts as gatekeeper to the more specialised sectors of health care. Access to psychiatric services or other specialised mental health services is not possible without referral by a GP. In this respect, the situation is more or less comparable to the UK or Denmark, but far less so to the USA, for example.

### Comparison with existing literature

It seems that patients with the most severe anxiety or depression symptoms have the highest chance of being diagnosed and treated by their GP. This has also been found in previous studies ([25,41-44]and [45]).

Severity of symptoms was strongly associated with poorer clinical outcome after one year, a finding which has also emerged in other studies [25,46,47]. We did not find significant associations between guideline adherence and prognosis, as was the case in [25], although Simon et al [46] reported a significant reduction in symptoms among recognised cases compared with non-recognised cases after 3 months, a difference that had disappeared after 12 months.

Based on the literature, one would expect better diagnosis combined with worse prognosis for more severe depression. A 10-year follow-up study found that even though treatment for depressed primary care patients was 'inadequate by psychiatric standards', the majority of the patients had a favourable outcome without recurrences [48]. There may be good reasons why GPs deviate from the guidelines, and patients get better regardless of whether their treatment is in accordance with clinical guidelines.

Regarding patient characteristics, in line with earlier studies [25,29], we also found that people who are more highly educated and in work are more likely to have a better outcome than people who are lower educated and unemployed. We can also confirm associations of clinical outcome with social support [27,29] and comorbidity of anxiety and depressive disorders [32] found in previous studies.

## Conclusion

Guideline concordant care is provided by general practitioners for more severe cases of depression and anxiety. Less severe cases improve just as much without guideline concordant care. In discussions on the introduction of DSM-V, some experts have argued that mental disorders in general and depression in particular have been defined too broadly. Lichtenberg and Belmaker [49] made an intuition based proposal for classifying types of depression heuristically, which is adopted by Bech [50], who distinguished primary depression (melancholia) from depression that is secondary to stress and depression that is secondary to medical conditions (post-natal depression, post-stroke depression, substance abuse disorder). Primary depression and depression secondary to medical conditions show good dose-response reactions to medication, which is less clear in the case of depression secondary to stress. It is possible that GPs are more sensitive to the first and miss the latter, from which there is a better spontaneous recovery. This might explain our results.

### Implications for future research or clinical practice

Our findings have some practical implications, as well as implications for future research. GPs tend to follow clinical guidelines more closely when managing depression and anxiety when patients have more severe symptoms. However, GPs could give more attention to lower educated patients with a small personal network, and to those who are unemployed. Finally, since patients who do not receive guideline concordant care seem to improve just as much as those who received guideline concordant care, further research is needed to establish the precise reason for this. A possible line of research might be a further elaboration of the distinction between primary depression to which the guidelines should be applied, and stress induced depression and anxiety, to which a more detached attitude might be desirable.

## Competing interests

The authors declare that they have no competing interests.

## Authors' contributions

MP took part in data collection in GPs' medical records, analysed the data and wrote the first draft.

PV participated in the original design of the NESDA study, designed this particular study and obtained funding for it, supervised analysis and writing and wrote the final draft. MS took part in data collection from GPs' medical records and commented on each of the drafts. PS did the multilevel statistical analysis and commented on several drafts. ML collaborated in the design of this particular study and commented on several drafts.

KvdM participated in NESDA, supervised MP's work as one of her PhD supervisors and commented on several drafts. HvM participated in NESDA and commented on several drafts. BP is principal investigator of NESDA and commented on several drafts. JB supervised MP's work as first supervisors of her PhD and commented on several drafts.

All authors read and approved the final manuscript.

## Pre-publication history

The pre-publication history for this paper can be accessed here:

http://www.biomedcentral.com/1471-244X/11/180/prepub
